# Automated Task-Transfer Function Measurement for CT Image Quality Assessment Based on AAPM TG 233

**DOI:** 10.3390/jimaging11080277

**Published:** 2025-08-18

**Authors:** Choirul Anam, Riska Amilia, Ariij Naufal, Eko Hidayanto, Heri Sutanto, Lukmanda E. Lubis, Toshioh Fujibuchi, Geoff Dougherty

**Affiliations:** 1 Department of Physics, Faculty of Sciences and Mathematics, Diponegoro University, Semarang 50275, Indonesia; riskamilia0@gmail.com (R.A.); ariij.2019@fisika.fsm.undip.ac.id (A.N.); ekohidayanto@fisika.fsm.undip.ac.id (E.H.); herisutanto@live.undip.ac.id (H.S.); 2 Department of Physics, Faculty of Mathematics and Natural Sciences, Universitas Indonesia, Depok 16424, Indonesia; lukmanda.evan@sci.ui.ac.id; 3 Department of Health Sciences, Faculty of Medical Sciences, Kyushu University, Fukuoka 812-8582, Japan; fujibuchi.toshioh.294@m.kyushu-u.ac.jp; 4 Applied Physics and Medical Imaging, California State University Channel Islands, Camarillo, CA 93012, USA; geoff.dougherty@csuci.edu

**Keywords:** task-transfer function, spatial resolution, ImQuest, iQmetrix-CT, IndoQCT

## Abstract

This study aims to develop and validate software for the automatic measurement of the task-transfer function (TTF) based on the American Association of Physicists in Medicine (AAPM) Task Group (TG) 233. The software consists of two main stages: automatic placement of the region of interest (ROI) within circular objects of the phantoms and calculating the TTF. The software was developed on four CT phantom types: computational phantom, ACR 464 CT phantom, AAPM CT phantom, and Catphan^®^ 604 phantom. Each phantom was tested with varying parameters, including spatial resolution level, slice thickness, and image reconstruction technique. The results of TTF were compared with manual measurements performed using ImQuest version 7.3.01 and iQmetix-CT version v1.2. The software successfully located ROIs at all circular objects within each phantom and measured accurate TTF with various contrast-to-noise ratios (CNRs) of all phantoms. The TTF results were comparable to those obtained with ImQuest and iQmetrix-CT. It was found that the TTF curves produced by the software are smoother than those produced by ImQuest. An algorithm for the automated measurement of TTF was successfully developed and validated. TTF measurement with our software is highly user-friendly, requiring only a single click from the user.

## 1. Introduction

The rapid advancement of computed tomography (CT) technology and its critical role in numerous diagnostic applications necessitate ongoing optimization of its performance [1,2]. A notable trend in CT development, particularly in CT reconstruction such as iterative reconstruction (IR) and deep learning iterative reconstruction (DLIR) techniques, is to continuously refine an approach for quality control (QC) procedures [3,4]. This new approach complements traditional approaches. Among the QC parameters for evaluating CT performance is image spatial resolution.

Spatial resolution describes the level of sharpness of an image. Spatial resolution is typically characterized by the modulation-transfer function (MTF), which quantifies the system’s response across spatial frequencies of interest. The MTF curve provides insights into the CT system’s ability to render sharp image edges and discernible object details at specific spatial frequencies, often summarized by the frequencies at 50% and 10% of the response (i.e., MTF at 50% and 10%) [5,6,7]. In stationary systems, such as those employing filtered-back projection (FBP) for image reconstruction, the system’s response characteristics remain consistent across all cross-sectional positions and contrasts, a property known as linear and shift-invariant (LSI) system [8,9]. However, with the advent of IR and DLIR techniques, the spatial resolution characteristics of CT systems behave non-linearly. The spatial resolution of IR and DLIR images depends on the local noise and contrast of the observed objects [10,11,12]. Hence, when images are reconstructed with IR and DLIR, the MTF is inadequate for assessing image spatial resolution.

The task-transfer function (TTF) is introduced to address the limitations of MTF. TTF takes into account information regarding local noise and contrast, typically expressed as the contrast-to-noise ratio (CNR) [13], to avoid over-generalizing the results to all features in the image [14,15,16]. The TTF is adopted by the American Association of Physicists in Medicine (AAPM) Task Group (TG) 233 [17]. Numerous studies have explored image quality assessment in IR and DLIR approaches [18,19,20,21], and reported that TTF provides a more comprehensive characterization of image spatial resolution across various contrast levels [11,16,22]. Measuring TTF typically requires a dedicated phantom containing objects with various contrast levels. These objects, often represented by pins of different densities and effective atomic numbers, are generally used to evaluate CT number linearity. Due to the complexity of measuring TTF across multiple objects simultaneously, a tool to streamline this process is essential.

Two examples of software, i.e., ImQuest and iQmetix-CT, were introduced [15,23,24]. Both of these employ the circular edge method to derive the edge spread function (ESF) from a circular disc. While imQuest generally produces accurate TTF results, its reliance on user-defined regions of interest (ROIs) introduces potential inter- and intra-variabilities [15,23]. In addition, imQuest measures one object at a time, whereas phantoms usually have several objects. Therefore, manual TTF measurements take a longer time. iQmetrix-CT performs measurements using pre-configured phantom templates [24]. Thus, measuring TTF using iQmetrx-CT is quite complicated. This limitation has motivated the development of a fully automated TTF measurement method on all objects within four available phantoms. By minimizing user intervention, a fully automated approach promises greater measurement accuracy and consistency, ultimately enhancing the efficiency of QC procedures.

## 2. Materials and Methods

### 2.1. Phantoms and Image Acquisition

**Figure 1:**
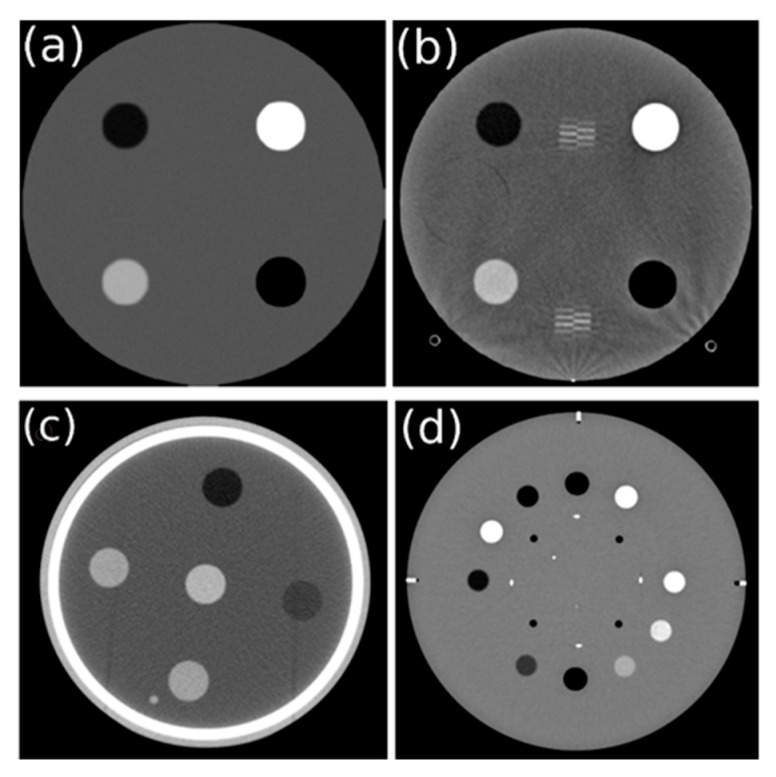
Sample images of four different phantoms: (**a**) Computational multi-pin phantom, (**b**) ACR 464 CT phantom, (**c**) AAPM CT phantom, and (**d**) Catphan^®^ 604 phantom.

We implemented our proposed method using four phantoms: the computational phantom, the ACR 464 CT phantom, AAPM CT phantom, and Catphan^®^ 604 phantom (Figure 1). The computational phantom images (Figure 1a) provide a controlled environment for evaluating the automated method, serving as idealized reference images [13]. The phantom was degraded with different spatial resolution: 0.4 lp/mm (low), 0.8 lp/mm (moderate), and 1.2 lp/mm (high). These images were generated with a diameter and field of view (FOV) of 200 mm. The phantom was designed similar to the ACR 464 CT phantom without the presence of objects for slice thickness measurement and ball-bearing objects on the edge for phantom alignment.

A sample image of the ACR 464 CT phantom for TTF measurement is shown in Figure 1b. The ACR 464 CT phantom images were acquired using the scan parameters detailed in Table 1. These images were reconstructed using both filtered-back projection (FBP) and adaptive statistical iterative reconstruction (ASiR) at 50% to demonstrate the differences in TTF measurements.

A sample image of the AAPM CT phantom for TTF measurement is shown in Figure 1c. The scan was performed using three different reconstruction filters: bone, soft tissue, and lung. Other exposure parameters remained constant, including a tube voltage of 120 kVp, tube current of 387 mA, slice thickness of 5 mm, revolution time of 0.75 s, FOV of 260 mm, and a pitch of 1.412.

The Catphan^®^ 604 phantom was scanned using three different deep learning image reconstruction (DLIR) algorithms: low (DLIR L), medium (DLIR M), and high (DLIR H). Other scan parameters were fixed, including a tube voltage of 120 kVp, tube current of 340 mA, slice thickness of 0.625 mm, revolution time of 1.0 s, and an FOV of 250 mm in axial mode. A sample image is shown in Figure 1d.

### 2.2. Automated TTF Measurement

**Figure 2:**
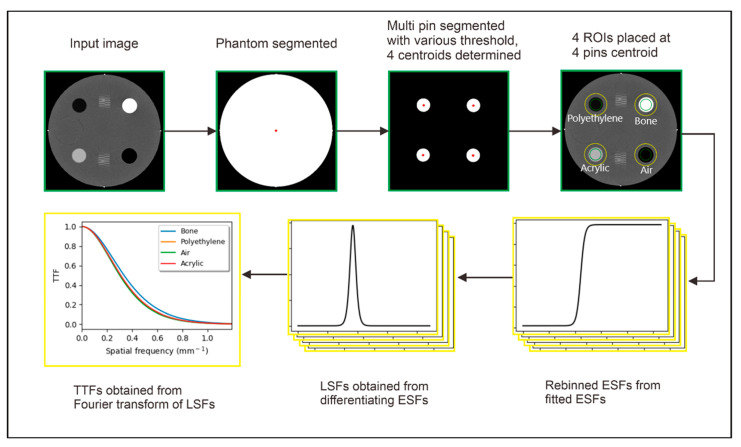
Automated TTF measurement process on the images of the computational and the ACR CT phantoms. The process was divided into two main stages: automatic ROI placement (green) and TTF calculation (yellow). The first process was started by opening the phantom image and segmenting it using a threshold of −200 HU. Multi-pins within the phantom were segmented using material-specific thresholds to determine their centroid coordinates. The second process was started with extracting edge spread function (ESF) from four regions of interest (ROIs). ESF samples were obtained by recording pixel profiles radially from the center to the edge of each pin. The ESF samples were fitted and differentiated to obtain the line spread function (LSF), and the task-transfer function (TTF) was calculated by applying the Fast Fourier Transform (FFT) to the LSF. Note: Green color indicates inner ROI, and yellow color indicates outer ROI. ESF is obtained from the CT number profile from the inner ROI to the outer ROI.

The automated TTF measurement was based on the AAPM TG 233 [13]. The automated system was developed in Python 3.7.9 and its graphical user interface (GUI) was built using the PyQt5 toolkit. Steps of automated TTF calculation using a multi-pin image from the computational and ACR 464 CT phantoms are illustrated in Figure 2. The process was divided into two main stages: automatic ROI placement (green) and TTF calculation (yellow).

The automatic ROI placement stage was started by loading the computational phantom image and ACR CT phantom image of module #1, which contains multiple pins having different CT numbers. The image of the phantom was segmented using a threshold of −200 HU to generate a binary image, where the phantom was isolated as the foreground. This binary image served two purposes: (1) measuring the phantom’s diameter and (2) creating a mask for further processing. The mask was applied to the input image by performing an element-wise multiplication, setting the air outside the phantom to 0 HU.

Next, multi-pin segmentation was performed on the masked image using material-specific thresholds, as detailed in Table 2. For each segmented insert, a binary image representing the pin material was generated. The centroid coordinates of each pin were calculated from these binary images. Four ROIs were automatically placed at each centroid coordinate, with each ROI having a diameter equal to 1:10 of the phantom diameter. Finally, the average CT number inside and outside the pins was computed using these ROIs.

The second stage was automatic TTF measurement, which consists of several steps. From the four segmented ROIs, pixel profiles were extracted radially from the center to the outer edge of each pin. These profiles were used to obtain ESF samples for each material at various contrast levels. Following this, phase alignment was applied to the ESFs. The phase alignment method employed a statistical approach, which is both fast and flexible [25]. This step was crucial for preserving the integrity and accuracy of the pin edge response information. Once the phase-aligned samples were obtained, a single-logistic curve fit was applied to estimate the ESF and reduce noise, as described in Equation (1). This fitting process ensures a smooth and accurate representation of the ESF, which is essential for subsequent TTF calculations.

(1) $$ESF\left(x\right)=\frac{a}{1+exp\left\{-b(x-c)\right\}}+d$$

The parameters *a*, *b*, *c*, and *d* in the single-logistic curve fit are determined using the non-linear least squares method, optimized with the Dogleg approach [26]. This results in fitted ESFs that are free of noise and ready for further processing.

Next, the four fitted ESFs were differentiated to obtain four line-spread functions (LSFs). These LSFs were then zeroed and normalized using standard methods. Finally, the task-transfer functions (TTFs) for each material were derived by applying the Fast Fourier Transform (FFT) to the four LSFs, as described in Equation (2):

(2) $$TTF\left(x\right)=\left|F\left(LSF\left(x\right)\right)\right|=\left|\int_{-\infty }^{+\infty }\left[LSF\left(x\right)e^{-2\pi jxf}\right]dx\right|$$

where F is Fast Fourier Transform, and f is spatial frequency.

**Figure 3:**
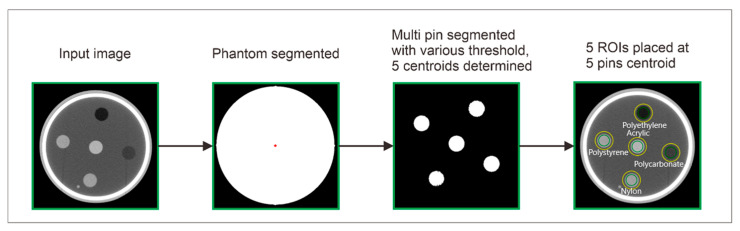
Automatic placement process for five ROIs on the AAPM CT phantom. Note: Green color indicates inner ROI, and yellow color indicates outer ROI. ESF is obtained from the CT number profile from the inner ROI to the outer ROI.

In principle, the TTF measurements for the other two phantoms follow the same procedure as described in Figure 2. However, the automatic ROI placement differs due to variations in the number, size, contrast, and positions of the multi-pins in each phantom. For the AAPM CT phantom, the automatic ROI placement is illustrated in Figure 3. The process was started by loading and segmenting the AAPM CT phantom image. The five pins were then segmented using material-specific thresholds, as outlined in Table 3. Once all pins were successfully segmented, the phantom proceeds to the TTF calculation stages.

**Figure 4:**
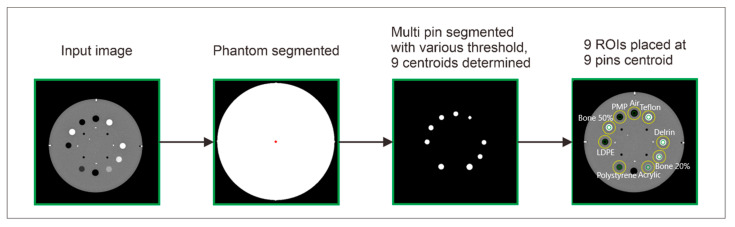
Automatic nine ROI placement process on the Catphan^®^ 604 phantom. Note: Green color indicates inner ROI, and yellow color indicates outer ROI. ESF is obtained from the CT number profile from the inner ROI to the outer ROI.

The difference in automatic ROI placement was also conducted in the Catphan^®^ 604 phantom, as illustrated in Figure 4. The process was started by inputting the Catphan^®^ 604 phantom image and segmenting it using a threshold of −200 HU. Following this, the nine pin objects within the phantom were segmented using the threshold values provided in Table 4. This segmentation process generated the centroid coordinates for each object, enabling the placement of ROIs on each pin for further analysis.

**Figure 5:**
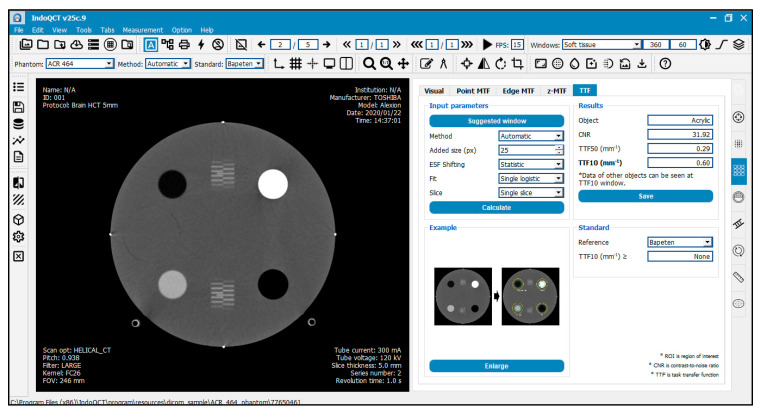
Graphical user interface (GUI) of the automated TTF measurement. The GUI allows users to configure the ESF sample length by adjusting the “Added size” parameter. Additional options, such as ESF shifting and curve fitting, can be enabled to optimize the results.

This automated algorithm is integrated in the IndoQCT software [27], designed to measure CT image quality, particularly in terms of spatial resolution. The graphical user interface (GUI) of IndoQCT is shown in Figure 5, and the tool can be accessed via https://indosect.com/indoqct, accessed 1 July 2025.

### 2.3. Comparison with ImQuest and iQmetrix-CT

To verify the TTF measurement results of our software, we compared them with the measurement results of established tools, ImQuest (https://imquest.vm.duke.edu/, accessed on 1 July 2025) and iQmetrix-CT (https://github.com/SFPM/iQMetrix-CT, accessed on 1 July 2025) (as gold standards).

ImQuest requires manual placement of regions of interest (ROIs) on the object of interest via its graphical user interface. While the TTF curve and measurement results are immediately visible, the manual nature of ImQuest necessitates performing measurements manually on the phantoms used in this study.

iQmetrix-CT, on the other hand, performs measurements using pre-configured phantom templates. It provides templates for two specific phantoms: the ACR 464 and the Catphan 600©. For any other phantom, a new template must be created manually by the user, including defining the angle of each object. However, in this study, successful TTF measurements were performed only on the ACR 464 phantom.

### 2.4. Statistical Analysis

To validate our methodology, we performed statistical analysis using IBM SPSS Statistics 27.1.0.1 (IBM SPSS). Our goal was to see how well the new methods aligned with existing ones.

First, we performed correlation analysis to understand the relationship between the measurements. We set a 95% confidence level to evaluate if the results from our methods were synchronized. A strong correlation coefficient (r) close to +1.0 would tell us there was a perfect positive relationship, confirming the consistency of our measurements.

Next, we used Bland–Altman Agreement Analysis. This step was crucial for identifying any systematic differences. By plotting the data, we could calculate the average difference (bias) and the range of expected differences (limits of agreement), to see if one method consistently measured higher or lower than the other.

Finally, we performed the non-parametric test Mann–Whitney U test for two datasets and Kruskal–Wallis test for three datasets. This allowed us to determine if the average difference we observed was a genuine, statistically meaningful discrepancy or simply the result of random chance. We set our significance value at a *p*-value = 0.05, a common benchmark in research, to make this critical distinction. Together, these analyses gave us a comprehensive statistical picture of our methodology’s validity.

## 3. Results

### 3.1. TTF on Computational Phantom Images

**Figure 6:**
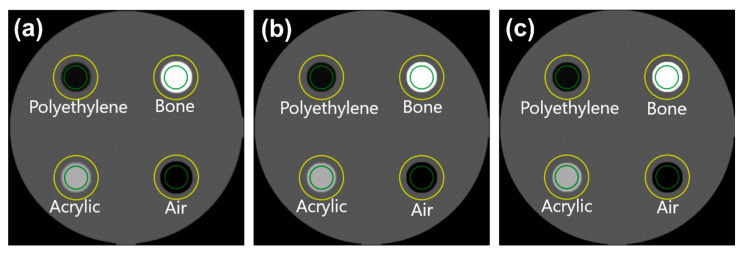
Automatic placement of ROIs on a computational phantom image at different spatial resolutions: (**a**) 0.4 lp/mm (low), (**b**) 0.8 lp/mm (moderate), and (**c**) 1.2 lp/mm (high). Note: Green color indicates inner ROI, and yellow color indicates outer ROI. ESF is obtained from the CT number profile from the inner ROI to the outer ROI.

**Figure 7:**
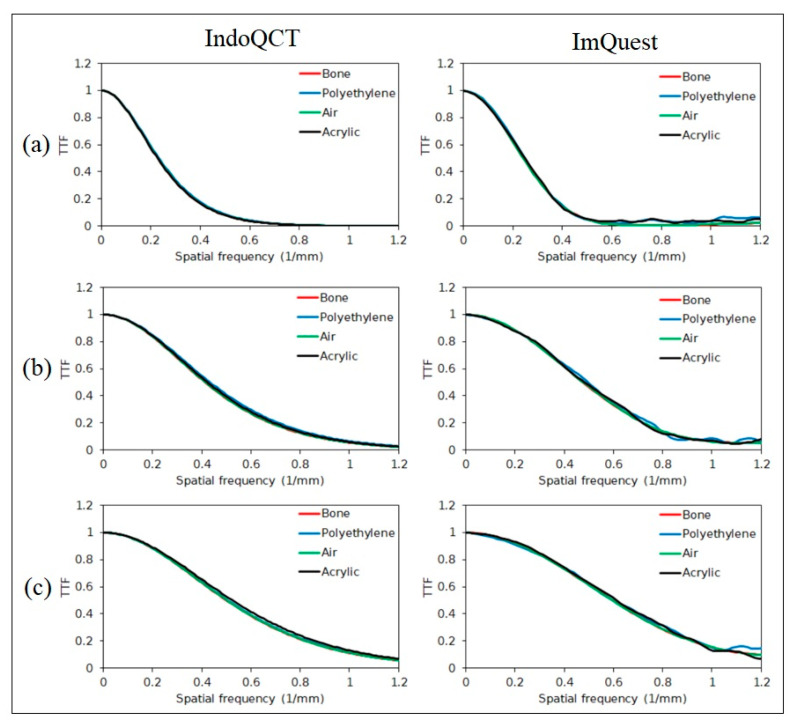
TTF curves of computational phantom images generated by IndoQCT and ImQuest at different spatial resolutions: (**a**) 0.4 lp/mm (low), (**b**) 0.8 lp/mm (moderate), and (**c**) 1.2 lp/mm (high).

Figure 6 displays the results of automatic ROI placement on computational phantom images at various spatial resolution levels. The segmentation method successfully achieved accurate ROI placement. Our software is capable of generating TTF curves at all spatial resolution levels. The results of TTF measurements on computational phantom images at different resolution levels, compared with ImQuest, are shown in Figure 7. It is clear that the TTF curves generated by our software are comparable to those by ImQuest. It is noted that the TTF curves by our software are smoother than those produced by ImQuest at low, medium, and high resolutions. Since the computational phantom images lack non-linear properties, the TTF curves were identical across all contrast levels, as expected. The corresponding 50% and 10% TTF values are presented in Table 5. The 50% and 10% TTF values from both measurement methods show no significant differences at low and moderate resolutions, with a maximum difference of 0.07 mm^−1^. However, at high resolution, a notable difference emerged, particularly for polyethylene, where ImQuest yielded a 10% TTF value of 1.55 mm^−1^. It is important to note that the TTF curves generated by ImQuest exhibit fluctuations, which can lead to inaccuracies in the 10% TTF values. In this case, a false value was observed for polyethylene.

The statistical analysis revealed a strong positive correlation between the two software results, with coefficients of 0.997 for CNR, 0.988 for 50% TTF, and 0.932 for 10% TTF. The Bland–Altman analysis showed that IndoQCT consistently produced lower results compared to ImQuest. The 95% limits of agreement were [−14.15, 4.97] for CNR, [−0.121, 0.008] for 50% TTF, and [−0.386, 0.279] for 10% TTF. The Mann–Whitney U test indicated that while the CNR and 10% TTF measurements were not statistically different (*p* > 0.05); however, the difference for 50% TTF was statistically significant (*p* = 0.03).

### 3.2. TTF on ACR 464 CT Phantom Images

**Figure 8:**
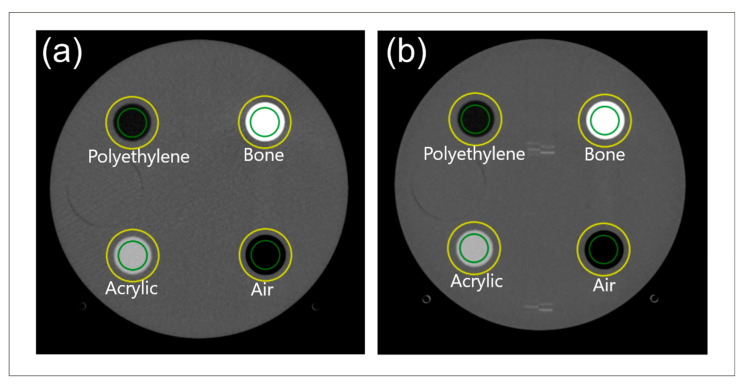
Automatic ROI placement on the ACR 464 CT phantom image at two different image reconstructions: (**a**) FBP and (**b**) ASiR. Note: Green color indicates inner ROI, and yellow color indicates outer ROI. ESF is obtained from the CT number profile from the inner ROI to the outer ROI.

**Figure 9:**
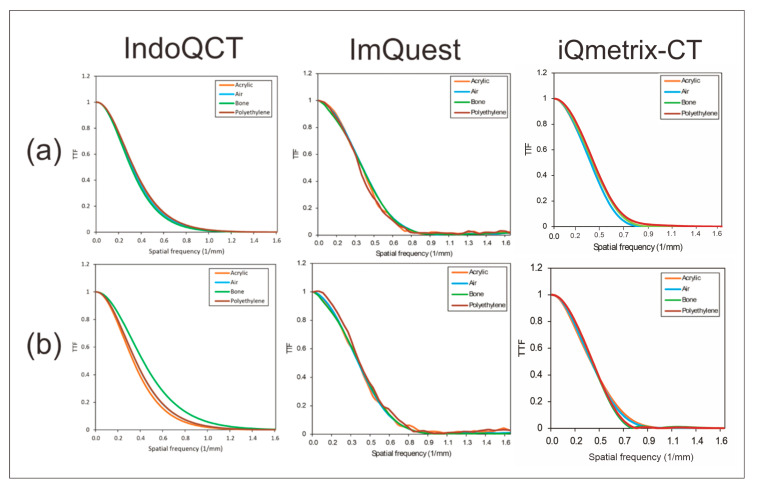
TTF curves of the ACR 464 CT phantom images generated by IndoQCT and ImQuest at two different image reconstructions: (**a**) FBP and (**b**) ASiR.

Automatic ROI placement on multi-pin objects of ACR 464 CT phantom images reconstructed using both FBP and ASiR was successfully carried out, as shown in Figure 8. The TTF curves generated by the two methods are shown in Figure 9, with the corresponding 50% and 10% TTF values summarized in Table 6. Overall, the TTF curves produced by IndoQCT are comparable and as stable as those from iQmetrix-CT. Even though the results from ImQuest are also considered comparable, the TTF curve from ImQuest fluctuates significantly. In FBP reconstruction, the TTF curves for all materials are similar, with a maximum difference of 0.07 mm^−1^ in the 10% TTF values. This indicates consistent spatial resolution across various contrast levels. By contrast, ASiR reconstruction results in more varied TTF curves, particularly for bone, which exhibits a higher response across all spatial frequencies. This phenomenon is observed in the TTF curves generated by IndoQCT, ImQuest, and iQmetrix-CT.

The statistical analysis showed a very strong correlation was only found on CNR measurements while the TTF measurements were weakly correlated and not statistically significant. The Bland–Altman analysis also found that despite the high correlation, the bias for CNR is very large when comparing iQmetrix-CT to the other two software, indicating iQmetrix-CT produces higher CNR values on average. The bias for TTF metrics was comparatively small across all pairs.

Meanwhile, the Kruskal–Wallis test showed other trends. A *p*-value > 0.05 was found from CNR and 10% TTF, which indicate that there is no statistically significant difference among the three methods. Despite this trend, the 50% TTF differs significantly with *p* < 0.05.

### 3.3. TTF on the AAPM CT Phantom Image

**Figure 10:**
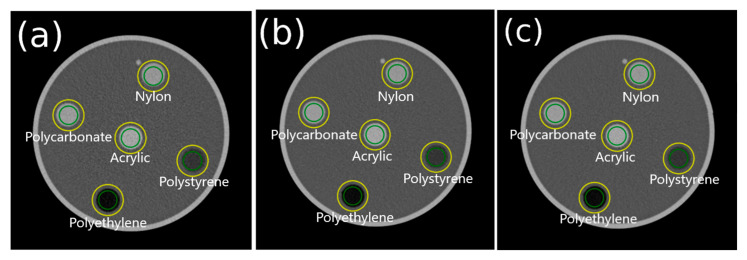
Automatic ROI placement on the AAPM CT phantom image at different slice thicknesses: (**a**) 2.5 mm, (**b**) 5 mm, and (**c**) 7.5 mm. Note: Green color indicates inner ROI, and yellow color indicates outer ROI. ESF is obtained from the CT number profile from the inner ROI to the outer ROI.

**Figure 11:**
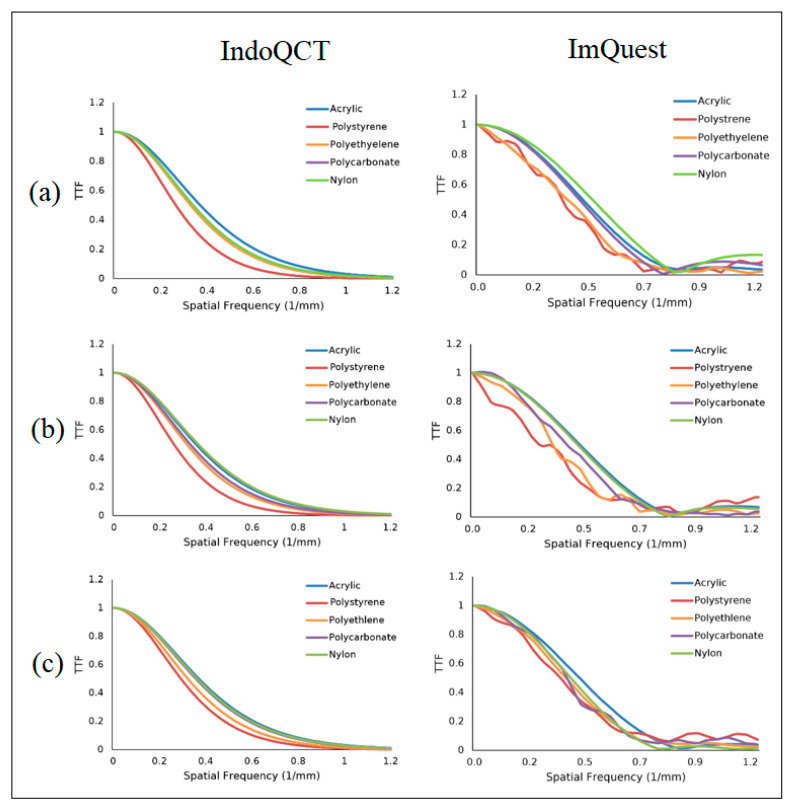
TTF curves of AAPM CT phantom images from IndoQCT and ImQuest at different slice thicknesses: (**a**) 2.5 mm, (**b**) 5 mm, and (**c**) 7.5 mm.

The algorithm successfully placed the ROIs on the AAPM CT phantom for various slice thicknesses, as shown in Figure 10. Figure 11 shows the TTF curves produced by IndoQCT and ImQuest, while Table 7 presents the numerical results for TTF 50% and TTF 10%. The TTF curves from IndoQCT exhibit more stability than those from ImQuest, particularly for low-contrast objects like polystyrene. Additionally, the TTF values remain relatively constant, as CNR increases only slightly with greater slice thickness.

The statistical analysis of the AAPM CT phantom revealed that the two methods lacked a strong positive correlation for both the CNR and TTF measurements. Furthermore, the results from IndoQCT were consistently lower than those from ImQuest, with narrow limits of agreement for 50% TTF [−0.174, 0.092] and 10% TTF [−0.087, 0.149]. The Mann–Whitney U test confirmed that the differences between the two methods were not statistically significant (*p* > 0.05) for CNR and 10% TTF comparisons, even though the 50% TTF yield *p* = 0.04 (indicating the statistical difference).

### 3.4. TTF on Catphan^®^ 604 Phantom Images

**Figure 12:**
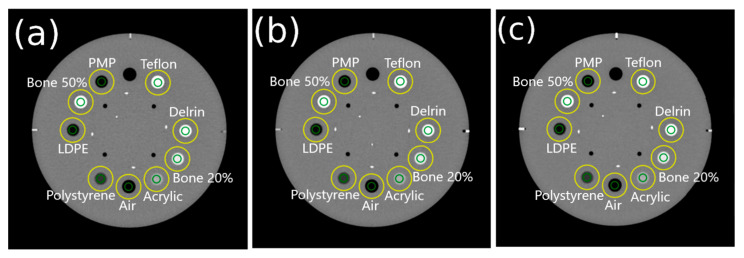
Automatic ROI placement on Catphan^®^ 604 phantom images for different DLIR levels: (**a**) low, (**b**) medium, and (**c**) high. Note: Green color indicates inner ROI, and yellow color indicates outer ROI. ESF is obtained from the CT number profile from the inner ROI to the outer ROI.

**Figure 13:**
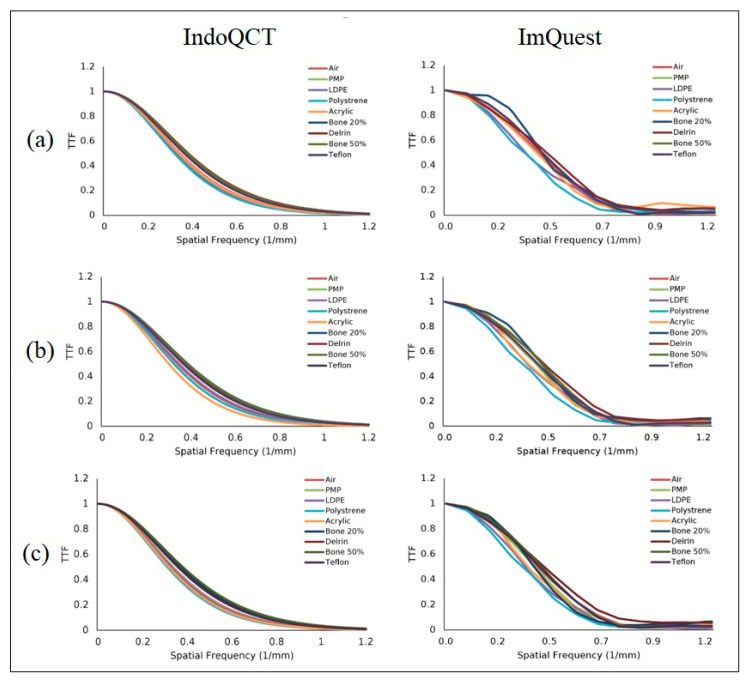
TTF curves of Catphan^®^ 604 phantom images from IndoQCT and ImQuest at different DLIR levels: (**a**) low, (**b**) medium, and (**c**) high.

Figure 12 shows the automatic ROI placement on the Catphan^®^ 604 phantom at various DLIR levels, demonstrating accurate placement. Figure 13 shows the TTF curves from ImQuest and IndoQCT, showing that IndoQCT produces more stable curves, particularly for high-contrast objects (Bone 50%, Bone 20%, Delrin, and Teflon). Table 8 presents the numerical results for TTF 50% and TTF 10%. It also shows that increasing the DLIR level has minimal impact on spatial resolution, with the highest observed increase from low to high being approximately 0.01 mm^−1^.

A very strong positive correlation was shown in CNR results from both methods, even though a weak correlation was found for TTF measurement. IndoQCT measures the 50% TTF 0.041 mm^−1^ lower than ImQuest, while for 10% TTF, IndoQCT produces 0.031 mm^−1^ higher results. The Mann–Whitney U test revealed that there was no statistically significant difference between both software in measuring CNR and 10% TTF, while the results differed significantly for 50% TTF.

## 4. Discussion

A fully automated TTF measurement method based on the AAPM TG 233 on various CT phantoms, i.e., computational, ACR 464 CT, AAPM CT, and Catphan 604 phantoms, has been developed. This software facilitates a more objective and efficient evaluation process of measuring TTF. Since TTF measurement is performed automatically (in placing ROIs within the images of the phantoms), this approach can minimize inter- and intra-variabilities. The algorithm was tested on four phantoms, and the results were compared with those obtained using ImQuest and iQmetrix-CT.

Overall, our algorithm demonstrated accurate TTF measurements across the four phantoms. Even though in most cases, the TTF from IndoQCT had no strong relationship with those from ImQuest and iQmterix-CT, the significance test revealed that the measurement of CNR and 10% TTF were not statistically different. The TTF measurements from IndoQCT were comparable to those from ImQuest and iQmterix-CT, with IndoQCT results tending to be lower. IndoQCT and iQmetrix-CT employ the curve-fitting technique [25], which produces a smoother TTF curve. IndoQCT uses of single logistic fitting effectively eliminated monotonic noise. However, it is important to note that single logistic fitting is only suitable for monotonic noise. For non-monotonic noise, double logistic fitting is required to achieve optimal curve-fitting results. In this study, we exclusively used single logistic fitting, which may explain the residual fluctuations observed, likely due to the presence of non-monotonic noise.

Another finding of this study is that DLIR reconstruction maintains the spatial resolution of images across various parameter settings. The Catphan^®^ 604 image response remained unaffected by the DLIR level used.

This result differs from the previous study [28] which reported that DLIR produced greater TTF values than FBP. It is important to note that the performance of DLIR relies on AI, meaning that it is highly dependent on the training data and its outcome is less predictable. By contrast, IR reconstruction can degrade image contrast beyond a certain threshold, making the resulting image quality more difficult to predict.

Automated TTF measurement greatly facilitates the measurement of the detectability index (d’), which is an important parameter for dose optimization and testing of new imaging systems, both in software and hardware. It is known that detectability index measurement also requires noise-power spectrum (NPS) measurement. The next study will focus on developing software for automatic NPS and detectability index measurement.

This study has some limitations. First, the algorithm was tested on only four types of phantoms. Expanding testing to include dedicated vendor-specific phantoms would enhance the universality of the proposed software. Additionally, the software was evaluated using a limited set of parameters. Further testing with variations such as IR levels or reconstruction filters is necessary, as contrast degradation is likely to occur under these conditions.

## 5. Conclusions

An algorithm for the automatic calculation of TTF based on AAPM TG 233 has been successfully developed. It can be applied to all four phantoms used, viz. computational, ACR 464 CT, AAPM CT performance, and Catphan^®^ 604 phantoms. CNR and 10% TTF measurement using IndoQCT, ImQuest and iQMetrix-CT were not significantly different with *p* > 0.05 with a tendency of IndoQCT to yield a higher result. Measuring TTF with our software is very easy because it only requires one click from the user. The measurement results using our software are comparable to the results from ImQuest and iQMetrix-CT.

## Figures and Tables

**Table 1:** Scanning parameter for ACR 464 CT performance phantom in this study.

Parameter	FBP	ASIR 50
Scanner	Philips MX-16 slice	GE Revolution EVO
Tube current	300	160
Tube voltage	120	120
Slice thickness	5	5
Filter	SB	Head
Kernel	SB	Standard
FOV	222	235
Scan mode	Helical	Helical
Pitch	0.671	0.53

**Table 2:** Threshold used for multi-pin computational and ACR CT phantom segmentation.

Material	Estimated CT Number (HU) *	Lower Threshold (HU)	Upper Threshold (HU)
Bone	955	750	1300
Polyethylene	−95	−120	−70
Air	−1000	None	−800
Acrylic	120	100	150

* The value was obtained from the computational and ACR CT phantom instruction.

**Table 3:** Threshold used for segmentation of multi-pin AAPM CT phantom.

Material	Lower Threshold (HU)	Upper Threshold (HU)
Acrylic	80	165
Polycarbonate	100	114
Polyethylene	−110	−60
Polystyrene	−55	−20
Nylon	80	115

**Table 4:** Threshold used for segmentation of multi-pin Catphan^®^ 604 phantom.

Material	CT Number Range (HU)	Lower Threshold (HU)	Upper Threshold (HU)
Air	−1046 to −986	None	−900
Polymethyl pentene (PMP)	−220 to −172	−250	−160
Low-density polyethylene (LDPE)	−121 to −87	−121	−87
Polystyrene	−65 to −29	−65	−10
Acrylic	92 to 137	80	150
Bone 20%	211 to 263	200	280
Delrin	344 to 37	300	430
Bone 50%	667 to 783	600	800
Teflon	941 to 1060	900	None

**Table 5:** Measurements for 50% and 10% TTF on the computational phantom.

Spatial Resolution	Object	IndoQCT			ImQuest		
		CNR	50% TTF (mm^−1^)	10% TTF (mm^−1^)	CNR	50% TTF (mm^−1^)	10% TTF (mm^−1^)
Low	Bone	194.93	0.23	0.47	205.70	0.24	0.44
	Polyethylene	19.70	0.23	0.48	20.90	0.25	0.43
	Air	209.26	0.23	0.47	209.69	0.24	0.44
	Acrylic	24.59	0.23	0.47	25.90	0.25	0.43
Moderate	Bone	195.95	0.41	0.86	207.68	0.48	0.87
	Polyethylene	19.59	0.43	0.9	19.88	0.50	0.83
	Air	208.56	0.42	0.86	219.74	0.48	0.87
	Acrylic	24.70	0.42	0.87	26.05	0.48	0.86
High	Bone	198.54	0.50	1.03	205.39	0.60	1.19
	Polyethylene	19.92	0.51	1.04	20.10	0.59	1.55
	Air	209.23	0.50	1.04	218.4	0.59	1.17
	Acrylic	24.91	0.52	1.09	25.57	0.61	1.14

**Table 6:** Measurements for 50% and 10% TTF on the ACR 464 CT phantom.

Reconstruction	Object	IndoQCT			ImQuest			iQmetrix-CT		
		CNR	50% TTF (mm^−1^)	10% TTF (mm^−1^)	CNR	50% TTF (mm^−1^)	10% TTF (mm^−1^)	CNR	50% TTF (mm^−1^)	10% TTF (mm^−1^)
FBP	Bone	106.70	0.30	0.63	146.66	0.37	0.65	339.86	0.40	0.70
	PE	23.05	0.33	0.68	23.13	0.34	0.63	51.87	0.45	0.72
	Air	233.68	0.31	0.65	233.64	0.37	0.66	438.19	0.37	0.68
	Acrylic	29.31	0.32	0.66	30.13	0.37	0.63	63.83	0.40	0.71
ASiR 50	Bone	147.37	0.42	0.87	155.89	0.38	0.67	160.49	0.34	0.67
	PE	38.56	0.36	0.73	22.82	0.38	0.70	33.58	0.34	0.67
	Air	325.38	0.35	0.73	339.59	0.37	0.66	322.99	0.38	0.70
	Acrylic	46.44	0.33	0.69	38.67	0.37	0.66	42.65	0.37	0.73

**Table 7:** Measurements for 50% and 10% TTF on the AAPM CT phantom.

Slice Thickness	Object	IndoQCT			ImQuest		
		CNR	50% TTF (mm^−1^)	10% TTF (mm^−1^)	CNR	50% TTF (mm^−1^)	10% TTF (mm^−1^)
2.5 mm	Acrylic	9.21	0.38	0.73	9.55	0.45	0.73
	Polycarbonate	11.28	0.38	0.68	10.98	0.43	0.69
	Polyethylene	9.87	0.29	0.63	10.78	0.38	0.66
	Polystyrene	4.31	0.18	0.51	4.21	0.36	0.66
	Nylon	5.45	0.33	0.66	5.64	0.49	0.76
5.0 mm	Acrylic	13.10	0.36	0.73	13.75	0.73	0.45
	Polycarbonate	15.72	0.33	0.68	15.36	0.40	0.69
	Polyethylene	14.12	0.32	0.65	15.02	0.34	0.67
	Polystyrene	5.98	0.26	0.54	6.07	0.28	0.69
	Nylon	8.56	0.36	0.75	7.08	0.45	0.72
7.5 mm	Acrylic	13.90	0.37	0.76	14.15	0.45	0.74
	Polycarbonate	16.29	0.36	0.74	15.03	0.4	0.67
	Polyethylene	15.21	0.32	0.67	13.91	0.39	0.68
	Polystyrene	6.23	0.29	0.60	6.29	0.37	0.75
	Nylon	8.72	0.36	0.75	5.56	0.41	0.67

**Table 8:** Measurements for 50% and 10% TTF on Catphan^®^ 604 phantoms for different DLIR levels of low, medium, and high.

DLIR	Object	IndoQCT			ImQuest		
		CNR	50% TTF (mm^−1^)	10% TTF (mm^−1^)	CNR	50% TTF (mm^−1^)	10% TTF (mm^−1^)
Low	Air	213.1	0.34	0.70	203.37	0.42	0.70
	PMP	41.44	0.33	0.69	45.38	0.41	0.70
	LDPE	28.53	0.34	0.70	30.44	0.41	0.71
	Polystyrene	19.22	0.32	0.66	17.70	0.33	0.63
	Acrylic	11.89	0.31	0.64	12.19	0.38	0.68
	Bone 20%	22.38	0.39	0.80	30.74	0.37	0.62
	Delrin	45.13	0.37	0.78	53.98	0.42	0.77
	Bone 50%	74.65	0.38	0.79	130.01	0.41	0.70
	Teflon	116.7	0.36	0.74	161.12	0.43	0.70
Medium	Air	263.6	0.34	0.70	245.53	0.42	0.71
	PMP	48.43	0.34	0.69	58.19	0.39	0.68
	LDPE	33.16	0.33	0.68	39.12	0.38	0.68
	Polystyrene	22.63	0.31	0.65	20.94	0.35	0.67
	Acrylic	14.62	0.32	0.67	13.69	0.42	0.73
	Bone 20%	23.37	0.38	0.78	36.40	0.43	0.70
	Delrin	53.31	0.38	0.78	66.14	0.43	0.75
	Bone 50%	69.48	0.38	0.78	145.97	0.44	0.71
	Teflon	141.4	0.36	0.74	190.09	0.43	0.70
High	Air	346.80	0.34	0.69	287.00	0.41	0.70
	PMP	62.73	0.33	0.69	71.49	0.40	0.68
	LDPE	41.74	0.33	0.68	42.45	0.30	0.50
	Polystyrene	28.59	0.31	0.65	30.55	0.29	0.51
	Acrylic	17.79	0.32	0.67	21.20	0.43	0.50
	Bone 20%	27.9	0.37	0.77	44.23	0.30	0.51
	Delrin	69.42	0.38	0.78	74.90	0.40	0.74
	Bone 50%	76.28	0.38	0.78	197.74	0.42	0.69
	Teflon	155.1	0.36	0.74	236.41	0.43	0.69

## Data Availability

The original contributions presented in this study are included in the article. Further inquiries can be directed to the corresponding author.

## Abbreviations

AAPM	American Association of Physicists in Medicine
ASiR	Adaptive Statistical Iterative Reconstruction
CNR	Contrast-to-Noise Ratio
CT	Computed Tomography
DLIR	Deep Learning Image Reconstruction
ESF	Edge Spread Function
FBP	Filtered-Back Projection
FFT	Fast Fourier Transform
HU	Hounsfield Unit
IR	Iterative Reconstruction
LSF	Line Spread Function
LSI	Linear Shift Invariant
MTF	Modulation Transfer Function
ROI	Region-of-Interest
TTF	Task-Transfer Function
